# Platelet-rich plasma in patients affected with Peyronie’s disease

**DOI:** 10.1080/2090598X.2022.2135284

**Published:** 2022-10-25

**Authors:** Chatar Achraf, Pr Ammani Abdelghani, Pr El Anzaoui Jihad

**Affiliations:** a Urology department, CHU Hassan II, Fes, Morocco; b Military Hospital Moulay Ismail, Meknes, Morocco

**Keywords:** Peyronie’s disease, Platelet-rich-plasma, autologous therapy

## Abstract

**Objectives:**

The objective of our study is to discover and evaluate the effects of repeated intralesional injections inside the tunica albuginea of platelet-rich plasma (PRP) in the treatment of Peyronie’s disease (PD).

**Methods:**

As part of a prospective study over 12 months from February 2020 until February 2021, on Sixty-five patients with Peyronie’s disease, and penile curvature between 25 and 45°. Patients were stratified into two groups, the first with a curvature between 25 and 35° and the second between 35 and 45°. Gathered data included patient-demographics, Injection technique, outcomes: both quantitative (curvature assessments) and qualitative (state of erectile function, pain during intercourse), and complications.

**Results:**

Patients in both groups received an average of 6.1 injections of PRP during the study period. Angulation was significantly improved in both groups an average final improvement of 16.88° (SD = 3.35) (p < 0.001) in the first group and 17.27° (SD = 4.22) (p < 0.001) in the second group. Pain during sex decreased from 70.7% to 34.25%, and 55.5% of patients had easier sexual intercourse.

**Conclusions:**

The positive results of our series of treatment for Peyronie’s disease by injection of platelet-rich plasma are encouraging both methodologically (simplicity) and clinical (safety and efficacy) as well as patient satisfaction.

## Introduction

Peyronie’s disease (PD) is a benign inflammatory disease of the penis, which results in the formation of localized scarring fibrous plaques of the albuginea causing a curvature with shortening of the penis during erections with painful erections.

The cause of Peyronie’s disease is not fully understood [1], but several factors appear to be involved.

A minor albuginea injury does not always lead to Peyronie’s disease. However, multiple factors can contribute to poor wound healing, scar tissue buildup, and fibrosis formation that could play a role in Peyronie’s disease [1].

These injuries can occur during sex (such as bending the penis during penetration or pressure from a partner’s pubic bone), sports activity, or following an accident. However, often, no specific trauma to the penis is reported.

It is often associated with penile pain, reduced penis length, erectile dysfunction threatening the psychological well-being of the couple [2], with loss of self-esteem and confidence.

PD usually occurs from the fifth decade but can occur at any age [3]. Its prevalence varies according to the different studies ranging from 0.5 to 9% in the general population [3,4].

In the management of Peyronie’s disease in its stable phase, a distinction is made between pharmacological and surgical approaches, depending on the severity of the penile deformity [5] and associated symptoms.

Surgical treatment is reserved for patients with severe penile curvature [6]. Hindering any sexual intercourse, surgery remains the fast and reliable treatment option.

However, there are emerging less-invasive treatments with controversial so-called “regenerative” use, such as extracorporeal shock wave therapy (Li-ESWT), intracavernous stem cell therapy (SCT) and platelet-rich plasma (PRP) injections [7].

Platelet Rich Plasma is an autologous biological therapy [8] containing a supraphysiological concentration of platelets, proteins, growth factors (derived from vascular endothelial growth factor (VEGF), platelets growth factor (PDGF), fibroblast growth factor (FGF), and transforming growth factor-β (TGF-β)); and other components of plasma that stimulate growth and repair in various target tissues. These growth factors affect stem cells recruitment, inflammatory reaction response, angiogenesis, and wound healing.

The use of PRP has affected all medical fields in hematology, rheumatology (treatment of tendinopathies) dermatology (treatment of alopecia, acne, and burns); maxillofacial surgery (degenerative joint disorders); and orthopedic [9–11].

PRP has received Food and Drugs Administration (FDA) approval for orthopedic bone transplantation [12]. Other uses are considered ‘off-label’ uses in urology.

In the urological sector, PRP, although still in its infancy, has been used mainly in the treatment of erectile dysfunction, Peyronie’s disease.

Autologous injections of PRP in the treatment of Peyronie’s disease is safe. In most studies [13,14] only minor side effects classified as grade 1 according to the Clavien-Dindo classification were reported. No major side effects or complications were noted.

In line with AAU and EAU recommendations [15,16], PRP has promising potential yet to be proven. The properties and physiological effects of PRP remain unknown and controversial in the context of autologous injections. A better understanding of this emerging controversial modality is essential to develop an effective management in the treatment of Peyronie’s disease.

In this article, we describe the evidence regarding the effectiveness and the safety profile of autologous PRP injections in the treatment of Peyronie’s disease. With the main endpoint, the improvement in curvature of the penis towards the midline at the end of treatment and a secondary endpoint which is the improvement in the penile pain score (numerical evaluation scales), the improvement in erectile function (the IIEF questionnaire5).

## Patients and methods

### Study design and population

As part of a prospective, open label, conducted at a single site in the Moroccan Kingdom over 12 months from February 2020 until February 2021.

The study included sixty-five patients with PD (n = 65).

Our inclusion criteria: naive patients who have not received any treatment for their Peyronie’s disease previously, with a stable symptomatic PD, having acquired angulation of the penis of at least 25 degrees in the dorsal, lateral, or dorsolateral regions plan, with an albuginea plate palpated on clinical examination (plate older than 6 months).

Exclusion criteria included curvature of the penis less than 25 degrees or more than 45 degrees on screening; history of spontaneous priapism; calcified plaque that would prevent proper administration of PRP; receiving previous oral or intralesional medical treatments for the PD.

Informed consent was obtained, and patients were aware use of an off-label treatment.

Ethical approval was obtained from the local ethics committee in the Military hospital of Meknes- Morocco and All patients consented to participate in the study and their information was handled as anonymous according to the ethical standards.

### Procedures

Once we selected our patients, given the absence of a control group and to assess the effectiveness of PRP and have a comparative study. We decided to divide our patients into two groups based on the degree of curvature, so we will have a view of the effectiveness of PRP as a function of the degree of curvature. We separated our patients into two groups according to the degree of penile angulation: the first group with an angulation between 25° and 35° and the tenth group with an angulation between 35° and 45°.

### Measurements

- The degree of angulation is measured using a medical goniometer or a protractor. It is the angle that represents the distance of the glans from the pubic line after injection of prostaglandin, measured on two photos: superior allowing to see the deviation on the axial plane (right or left) and in profile allowing to see the deviation on the sagittal plane (superior or inferior). This can be done at home: by a self-photograph of an erection obtained naturally (preferably) or by intra-cavernous injection of a vasoactive agent (erection induced pharmacologically) [17].
- Pain during intercourse: Penile pain was evaluated by use of a conventional 11-point

Pain-scale (pain intensity visual analog scale: VAS) [18].

- Erectile dysfunction (ED): Self-evaluation of the erection was performed by all patients by use of the IIEF questionnaire [19].

### Preparation of PRP

**Figure 1.**
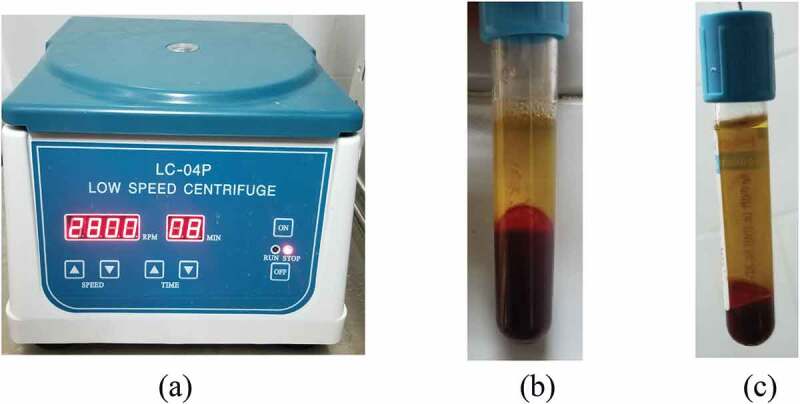
Photographs showing (a) centrifugation apparatus, (b) blood separation after ‘the first spin’ centrifugation and (c) platelet-rich plasma production after a ‘second spin’ to separate platelet-rich plasma from platelet-poor plasma.

We obtain the PRP following the centrifugation of 10 ml of the patient’s blood twice for 8 min at 2800 revolutions per minute (rpm) (Figure 1).

The first spin at 2800 rpm for 8 minutes was applied to the blood sample separating the platelet-poor plasma from the PRP and RBCs. The upper layer is removed and a second spin at 2800 rpm for 8 minutes separates the RBC fraction from the PRP.

After the second centrifugation, half of the surface layer of the plasma was removed leaving the platelet granules in the remaining half of the plasma [20].

According to current regulations, PRP must be prepared from fresh blood and injected during the same session.

We would like to add an important point, we must ensure that the centrifugation machine does not destroy the platelets; to our surprise we found counts without platelets because it was destroyed during the centrifugation.

During our studies, we performed several platelet counts after centrifugation to find the ideal setting (number of revolutions per minute) where we have the most platelets after centrifugation. in our case, the best output should be 2750 and 2850 rpm

### Injection of PRP

injection diagram: Our patients were subjected for two months to a session every 15 days, then at 3, 6, and 9 months.

Before treatment, all the patients underwent local anesthesia (dorsal nerve penile block) by 10 ml of 2% lidocaine injection at the base of the penis.

After performing a penile block, we inject 8 ml of PRP intra- and peri-lesion then at the level of the tunica *albuginea* of *corpora cavernosa* on a detumescent penis with a 21-gauge needle.

Then we compress the injection site to prevent bruising.

The check was carried out at the end of the protocol: the same parameters were evaluated: search for improvement in the angle of curvature, sexual satisfaction of the patient (evaluated with the IIEF5 questionnaire), plaque size, penis size, pain during intercourse sexual.

### Data collection

Demographic, clinical data: (angle of curvature, size of the plate, size of the penis, pain during intercourse, Erectile dysfunction (ED)).

The complication of the injections (using Clavien Dindo classification), procedure details, outcome data has been collected and evaluated after 12 months of follow-up.

The primary endpoint was the percentage of the degree of reduction in the change in curvature of the penis towards the midline at the end of treatment.

The secondary endpoint was the improvement in penile pain score (pain intensity visual analog scale), improvement in erectile function (the IIEF5 questionnaire).

### Statistical analysis

The data were presented in Excel® and then analyzed by the Epi Info® 7.2.5 software. The paired t test was used to compare mean differences between pre- and post-treatment IIEF-5 scores, also change in penile curvature, penile size, and penile pain during intercourse (change versus baseline). We proceeded to a descriptive analysis by establishing the means and standard deviations for the quantitative variables, the frequencies, and percentages for the qualitative variables.

A value of p < 0.05 was regarded as statistically significant.

## Results

The two groups of our study were comparable in terms of demographics and number (33 patients of the first against 32 patients of the second group). Both groups were in the stable phase of disease with plaque dating at least 6 months) (Table 1).

**Table 1.** Demographic distribution and patient’s characteristic.

Characteristic	First group angulation between 25–35 (n = 33)	Second group angulation between 35–45 (n = 32)
Age(y), mean (SD)	60.4 (10.2)	59.8 (9.9)
Penile size (cm), mean (SD)	10.08 (1.88)	10.22 (1.51)
Penile curvature (°), mean (SD)	31.39° (1.80)	39.1(2.74)
Direction of penile curvature, n (%)		
Right	13 (39.4)	14 (43.7)
Dorsal	8 (24.2)	10 (31.3)
Left	12 (36.3)	8 (25)
Penile plaque, n (%)		
1	29 (87.8)	24 (75)
2	3 (9.2)	5 (15.6)
>2	1 (3)	3 (9.4)
Erectile dysfunction, n (%)	22 (66)	26 (81)
Pain during intercourse, n (%)	22 (66)	24(75)
Number of injections, n (%)	6	6.3

Patients in the two groups received an average of 6.1 injections of PRP (range 4 to 7) during the study period.

All injections were well tolerated in all cases.

Checks are carried out, one, three, six, and nine months then annually, by measuring the evolution of deformities and especially the overall satisfaction of the patient and the improvement in his quality of life.

### Efficacy of PRP on peyronie’s desease

The mean curvature of the penis at baseline was 31.39° for the first group and 39.1° for the second group.

At the end of the last PRP injection, there was an improvement and decrease in the mean curvature of the penis from baseline in the first group of −16.88° (SD = 3.35) and the second group of −17.27° (SD = 4.22); the difference between groups = 0.3°, 95% CI = 1.26 to 1.37, P-value <0.001).

In addition to that, we noted an improvement in other symptoms (Table 2):

**Table 2.** Comparison of the results between the two groups before and after treatment with PRP.

	First group angulation between 25–35 (n = 33)		p-value	Second group angulation between 35–45 (n = 32)		p-value
	Before PRP	After the last injection of PRP		Before PRP	After the last injection of PRP	
Penile pain during intercourse (mean VAS, (mean SD))	4.1 (3.9)	2.5 (2.2)	0.0442	5.7 (4.4)	3.8(2.9)	0.0457
IIEF5 score (mean score, (mean SD)	6.3 (2.1)	7.6 (2.5)	0.0255	5.1 (2.5)	6.8 (3.2)	0.0210
Penile size	10.08 (1.88)	10.98 (2.04)	0.0670	10.22 (1.51)	11.1 (1.64)	0.0292

- The improvement in pain measured by visual analog scale (VAS) was markedly decreased in the two groups: first group −34% [SD = 2.2], for the second group −39% [SD = 2.9].

- The mean erectile function scores measured according to the IIEF score were improved compared to baseline for patients in the two groups: first group: mean change from the baseline before treatment: + 50% of patients [SD = 2.5] and for the second group + 61% [SD = 3.2].

- We also noted an increase in the size of the penis in the two groups: first group + 8.1% [SD = 2.04], and in the second group: + 7.92% [SD = 1.64]

#### Tolerance and side effects

In both groups, the injections were well tolerated.

Some complications were noted:

We had superficial hematoma following the injection of PRP in five patients.

Mild pain at the injection site in eight patients is classified as grade 1 according to the Clavien-Dindo classification (Table 3).

**Table 3.** Adverse effects and complication of PRP injection.

Events	First group angulation between 25–35°(n = 33)	Second group angulation between 35–45 (n = 32)
**Minor:**		
Mild pain	2 (6%)	6 (18%)
Bruising	2 (6%)	3 (9%)
**Major:**		
Bleeding	0	0
Infection	0	0
Compartment syndrome	0	0

No major side effects or complications were noted.

## Discussion

Our study of 65 patients confirms the positive conclusions of our preliminary trial.

Our main endpoint (degree of curvature) and our secondary endpoints (improvement in pain and erection) after the last PRP session (each patient had an average of 6.1 injections) showed significant improvement in PD lesion regression, assessed by curvature measurement, VAS pain questionnaire, and the IIEF-5 questionnaire.

An improvement in the angle of curvature was observed in both groups and a decrease in the mean curvature of the penis from baseline in the first group of −16.88° (SD = 3.35) and the second group of −17.27°.

Over the past five years, several studies have focused on the role of minimally invasive treatments of Peyronie’s Disease [21]. The therapeutic panel of Peyronie’s disease is very diversified. Beginning with oral treatments (analgesics, anti-fibrotic/antioxidants, and pro-erectile substances) which are more beneficial in the active phase of the disease all the way to more invasive intra-lesional injections or surgical treatments such as penile plication, plate excision/incision and grafting or implantation of a penis prosthesis.

There is also mechanical therapy (traction therapy, vacuum therapy) [22], which represents a conservative treatment to correct penile pain and support penis length.

New treatments have appeared in recent years. These less invasive and non-conventional therapies [23], included platelet-rich plasma (PRP), hyaluronic acid (HA), combination therapy of PRP and HA, extracorporeal shockwave therapy (ESWT), stem cell therapy (SCT), mycophenolate mofetil (MMF), and H-100.

Some of these minimally invasive therapies are still being tested, such as SCT which is a new treatment modality for PD [24], several studies have shown the role of SCT in human and animal models [25,26]. Also, MMF: has demonstrated its effectiveness in treating retroperitoneal fibrosis, by reducing the synthesis of collagen and the activity of fibroblasts [27]; Antoniassi et al. in 2019 showed no significant improvement after MMF in animal models [28]. Regarding the H-100: Levine et al. in 2016 [29] showed benefits in the treated group compared to the placebo group. ESWT is described by several studies which are based on mechanisms of action, which consist of the degradation of fibrous tissue by shock waves and the induction of the inflammatory response for lysis and elimination of plaque [30]; In the study by Claro et al. [31] and Di Mauro et al. [32] showed improvement in patients treated with ESWT. Concerning HA, which is an extracellular matrix glycosaminoglycan whose mechanism of action suggests that it acts against factors promoting inflammation and oxidative stress [33]; The two studies by Zucchi et al. [34] and Favilla et al. [35] report statistically significant improvements in various PD parameters, absolute changes may not be clinically appreciated.

PRP is an autologous therapy that may explain the safety and absence of side effects from its use. It is a concentrate of several growth factors (PDGF, TGF-β, IGF, EGF, VEGF) prepared following centrifugation of the patient’s blood [8]. The effects of intratunic injection of PRP for the treatment of PD in rats by Culha et al. [36] observed that PRP had no therapeutic effect on PD in the present study assessing the effects of PRP in the Peyronie model created with recombinant TGF-β1 injected into tunica albuginea of the rats.

Matz et al. [37] studied PRP injections in PD patients (n = 17), with an average of 2.1 injections per patient. International Index of Erectile Function (IIEF) scores increased by an average of 4.14 points for all PD patients with an 80% improvement in erectile function. 80% of patients reported a subjective improvement in their degree of curvature.

PRP has also been also combined with HA, this is reported in the study by Virag et al. [13] who studied the effect of a combination of PRP and HA for the treatment of PD with a large sample size (n = 90). They noted an improvement with a mean reduction of 18.08 degrees at 2 months, a decrease in their degradation, improvement in erections, and improved sexual activity. 73.3% of these ED patients demonstrated satisfactory results. The complications post-PRP intralesional injections included ecchymosis present in 16.7% of the patients and marked hematoma in 10%. Two patients suffered from transient hypotension after local anesthesia. No serious adverse events were recorded [13]. The limitations for the study by Virag et al.: the study lacked a control group, which made it difficult to obtain significant insight into this combination therapy versus to the current standard of care.

In intra-lesional injection therapy (Collagenase Clostridium Histolyticum, verapamil, IFNα2b), only Collagenase Clostridium Histolyticum has Marketing Authorization for Peyronie’s disease [38].

Despite these intralesional injection treatments, the natural course of the disease may continue. Meanwhile the various research concerning the physiopathology of this disease, its medical treatment is uncertain. No treatment has shown convincing efficacy. PRP has been proposed for PD without prospective randomized studies [16].

A comparison of our data with other trials of intra-lesional injections, such as that of Virag et Al. [13], shows similar results, although, in his study [39], Virag combined platelet-rich plasma and hyaluronic acid. Notsek et Al. [40] who divided his study population (n = 59) into 2 groups: first group (n = 32) intralesional PRP injections and the second (n = 27) intra-lesional injections of sodium chloride 0,9%.

The administration of PRP allowed for a decrease in the degree of penile curvature. Virag et al. [13], described a reduction of the curvature on average of 18.08° while Notsek et al [40], reported a decrease in curvature angle in 50% of patients from the first group and in 22.2% of patients from the second. However in our case series there was an average reduction of 17.07° for the two groups combined. In addition, the erectile dysfunction IIEF-5 score in patients who were recipients of the PRP intralesional injections decreased as shown in the study by Virag et al. [13], which described 43.3% of patients had an improvement in their IIEF-5 score while Notsek et al. [40], an improvement of 56.3% of patients in the group 1, whereas the percentage in the control group was significantly lower 3.7%. Our study showed a major improvement in IIEF-5 Score of 55.5% of our patients (our two groups combined).

Notsek et al. [40], described a pain reduction of 84% of the first group, while the second group in comparison had a low pain reduction rate of 29.6% of their second sample size. In our study, the improvement in pain was markedly decreased in the two groups combined −36.5%.

In our study, complications were limited to transient ecchymosis and mild pain even in patients on anticoagulant therapy.

Based on the use of extemporaneously treated autologous blood, PRP is an outpatient treatment that complies with all regulations concerning handling fresh blood. It is economical, reproducible, and easy to obtain (autologous blood), without significant complications.

However, our study lacked a control group, making it difficult to gain meaningful insight into this therapy compared to the current standard of care.

PRP is currently used in various specialties. Theoretically, this technique can be useful in various urological pathologies including ED, stress urinary incontinence, and PD.

Given its minimally invasive nature and compared to surgical treatments and their 20% risk of ED when it comes to patching incisions, PRP brings enormous hope as a future therapeutic agent.

## Conclusion

The short-term positive results of our case series for the treatment for Peyronie’s disease by injection of platelet-rich plasma are encouraging both methodologically and clinically. The patients as well were very satisfied with the favorable evolution of their disease. The results are encouraging and require further research in this direction.

## Abbreviations

HA: hyaluronic acid; IIEF: International Index of Erectile Function; Li-ESWT: extracorporeal shock wave therapy; MMF: mycophenolate mofetil; PD: Peyronie’s disease; PRP: platelet-rich plasma; RBCs: red blood cells; SCT: stem cell therapy; VAS: visual analog scale.
